# Primary Pleomorphic Liposarcoma of Liver: A Case Report and Review of the Literature

**DOI:** 10.1155/2013/398910

**Published:** 2013-01-10

**Authors:** P. R. Thippeswamy Naik, Prem Kumar, P. Vinod Kumar

**Affiliations:** Department of General Surgery, Victoria Hospital, Bangalore Medical College & Research Institute, Bangalore, Karnataka 560002, India

## Abstract

Primary liver liposarcoma is a rare disease. The knowledge of the clinical course, management, and prognosis of primary liver liposarcoma are all limited because of its rarity. Twelve cases of primary liposarcoma of the liver have been previously reported. We present the thirteenth case, which occurred in an adult male patient. A 42-year-old male patient came to our outpatient department with complaints of pain abdomen, mass per abdomen, and weight loss. Ultrasonography showed a mass arising from the the left lobe of liver. CT abdomen showed a heterogenous enhancing mass from left lobe of liver with multiple cystic and necrotic areas compressing the stomach and spleen with no evidence of metastasis. Differential diagnosis included adenoma and primary malignancy. Exploratory laparotomy and resection were done. HPE was found to be pleomorphic liposarcoma of liver.

## 1. Introduction

Virchow first described a malignant tumor of fatty tissue arising in the lower extremity in 1857 [1]. Since then, liposarcomas have been identified in a number of tissue and organs, but only rarely in the liver. The most common sites are the deep soft tissue of the trunk, retroperitoneum, and the subcutaneous fat of the upper and lower extremities [2]. Liposarcoma itself constitutes about 9.8% to 18% of soft tissue sarcomas; its incidence is second only to that of Malignant Fibrous Histiocytoma (MFH) [3]. The experience in dealing with this malignant neoplasm is limited because of its rarity. Primary liposarcoma of the liver must be considered in the differential diagnosis of a hepatic mass that develops in a noncirrhotic liver in whom liver transplantation may be considered as liposarcoma is clearly an absolute contraindication for liver transplantation [4]. Hepatic resections might be efficient although prognosis is poor and recurrence is more.

Furthermore, most of the articles describing liver liposarcoma concentrate only on histopathological and imaging studies. The clinical course and prognosis of primary liver sarcoma are rarely mentioned. Hence we report a case of liposarcoma liver in detail.

## 2. Case Report

**Figure 1:**
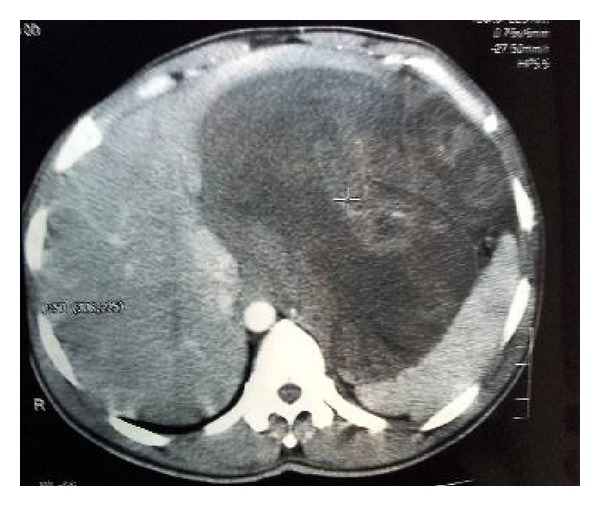
CECT Showing the huge mass from the left lobe of liver, compressing liver and spleen.

A 42-year-old male patient had history of pain abdomen which he attributed to a mass per abdomen noticed since 15 days. Pain was intermittent, dull aching variety with symptom-free intervals. He also complained of weight loss. On examination, a mass of size approximately 15 × 10 cm was felt occupying the epigastrium and left hypochondrium. All the blood tests were normal. Chest X ray revealed elevation of left hemidiaphragm and Ultrasonography revealed a large heterogenous mass lesion of size around 18 × 15 cm arising from the liver occupying the epigastrium, left hypochondrium extending up to splenic hilum. Liver function tests were normal. CT abdomen showed a large lobulated, well-defined, heterogeneously enhancing mass lesion of size 18 × 14 × 13 cm from the left lobe of liver with multiple cystic, necrotic areas and compressing the stomach and spleen (Figure 1). Upper GI Endoscopy showed external compression on the anterior wall of stomach.

**Figure 2:**
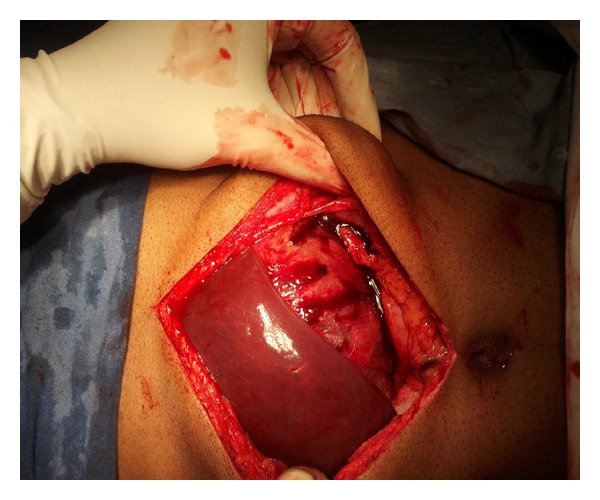
Liver mass seen on opening the abdomen.

Differential diagnosis of a primary hepatic adenoma and carcinoma was made. There were no risk factors for malignancy. AFP levels were normal. FNAC was not attempted due to risk of tumor seeding and haemorrhage. Metastatic workup was done and no metastases was identified. CT Chest, pelvis was normal. During laparotomy, mass was arising from liver (Figure 2). The left lobe of liver was thinned out because of the tumor pressure effect. The tumor was adherent to the left hemidiaphragm and the upper spleen.

**Figure 3:**
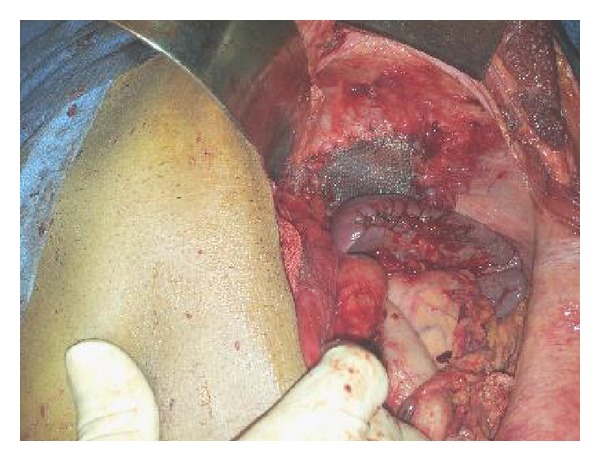
Postresection showing partially resected spleen and mesh repair of diaphragm.

En bloc resection of the tumor mass and extended left lateral hepatectomy with resection of the part of left hemi diaphragm and upper part of spleen was done. Diaphragmatic rent was closed with mesh and splenorrhaphy was done (Figure 3). 

**Figure 4:**
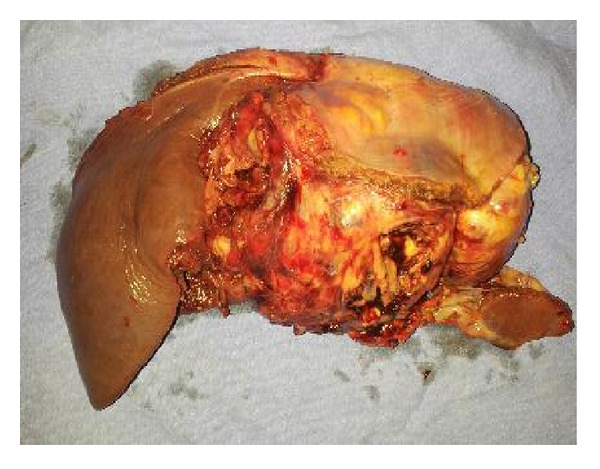
Gross specimen of tumor.

An ICD was also put in the left pleural space. Specimen was sent for histopathological department for examination. Grossly, cut section of the tumor showed a soft fleshy, friable mass with extensive foci of haemorrhage and necrosis (Figure 4).

**Figure 5:**
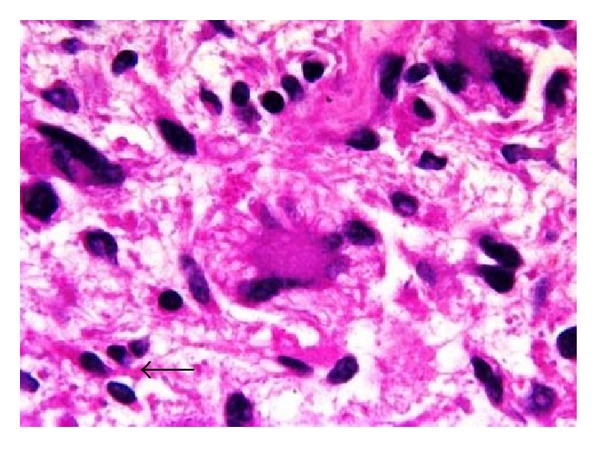
Microscopy showing large pleomorphic cells (arrow) interspersed in a loose connective tissue stroma.

Microscopy showed a tumor with sheets of large pleomorphic cells with vesicular hyperchromatic nuclei showing plenty of mitosis, occasional lipoblasts, and thick capsule with tumor infiltration suggestive of pleomorphic liposarcoma (Figure 5). Diaphragmatic tissue showed tumor remnants on the undersurface with focal infiltration and splenic tissue was normal with capsule showing infiltration.

*Immunohistochemistry*

Test result-NEGATIVE for S 100, CK, and EMA. Postoperative course was uneventful. The patient tolerated normal activity and enteral feeds. Chest tube was removed and discharged and he was referred to oncology department. Clinical stage: Stage III [T2b N0 M0 G3] as per AJCC staging. And hence the patient was put on adjuvant chemoradiotherapy and now is free of recurrence.

## 3. Discussion

Primary tumors arising from the liver are common. The differential diagnosis of primary hepatic mass should include Benign and Malignant tumors.

Classifications on the basis of cell origin include

- epithelial origin,
- mesenchymal origin,
- mixed origin,
- others.

Primary hepatic sarcomas are rare accounting for 1-2% of all primary malignant tumors of liver. Among these, angiosarcoma and hemangioendothelioma are most common [17]. Liposarcoma is a tumor derived from primitive cells that undergo adipose differentiation. It is largely a disease of adults, with peak incidence between 40 and 60 years age group, and it shows a slight predominance toward men [18]. When liposarcomas do occur in children, they tend to present in the second decade of life. In either event, the deep soft tissues of the extremities, particularly those of the thigh, are the most common location, accounting for more than 50% of liposarcomas [19]. One unique feature of liposarcoma is its tendency to occur in visceral spaces, particularly that of the retroperitoneum. Liposarcoma may occur in many other locations. About 5% occur in head and neck and 10% in upper extremity. Other unusual locations are axilla, vulva, peritoneal cavity, spermatic cord, and even the breast. 

There have been many attempts to classify liposarcomas depending on a combination of two basic histological aspects of the tumor:

- the stage of the differentiation of lipoblasts, based on relative amounts of lipid in the cells and myxoid material in the extracellular spaces, and
- the overall degree of cellularity and cellular pleomorphism.

Primary hepatic liposarcoma is extremely rare although liposarcoma is one of the common soft tissue malignancies which occurs usually in the extremities and retroperitoneum [20]. Early diagnosis of primary liposarcoma of liver is not easy. Liver is the largest solid organ in the body, and symptoms and signs of a tumor in the liver usually go undetected unless the tumors are large. The common symptoms and signs of primary liver liposarcoma include nausea, vomiting, fever, jaundice, abdominal fullness, right upper quadrant pain, and weight loss. Most of the symptoms are usually caused by displacement or compression of nerves, vessels, biliary tract, and intestines.

Abdominal ultrasonography usually is a good tool to do preoperative screening and postoperative followup. Computed tomography is still the best tool to evaluate the resectability of liver liposarcoma before surgery. Contrast enhancement depends on the level of differentiation. Little enhancement is noted in well-differentiated liposarcomas, and more with round cell, pleomorphic, and dedifferentiated subtypes. Corresponding heterogeneity with regards to contrast enhancement is seen in Myxoid variety [21]. Other findings of liposarcoma are thick fibrous septae, nodularity and foci of hemorrhage, and necrosis. Metastatic spread of soft tissue liposarcomas is relatively common, but still liver is involved in only 10% of cases. Metastases are usually found in the brain, pleura, thyroid, pancreas, and spinal cord.

*World Health Organization (WHO) Divides Primary Hepatic Liposarcomas into Four Subtypes Table 1 shows the four different WHO subtypes*

1. *Myxoid liposarcoma*: it is the most common type in pediatric age group. It is Intermediate grade but its round-cell variant is high grade in nature and can metastasize.
2. *Well-differentiated liposarcoma*: it is the most common subtype (50% of liposarcomas) and includes atypical lipoma. It is low grade (does not metastasize, but may recur locally). There is minimal risk of dedifferentiation.
3. *Dedifferentiated liposarcoma*: it is most common with retroperitoneal lesions. High-grade sarcoma arising in association with well-differentiated liposarcoma.
4. *Pleomorphic liposarcoma*: it is the rarest type (5–10% of liposarcomas). High grade in nature and may mimic MFH or even carcinoma or melanoma. There is very high chance of local recurrence and metastasis. (Compiled from Peterson et al. 2003 [3], Dei Tos 2000 [22], Coffin 1997 [19], Enzinger and Weiss 1995 [9], and Weiss and Rao 1992 [23])

A number of cytogenetic correlations also have been made with liposarcoma. Well-differentiated liposarcomas have been associated with abnormalities derived from the q13–15 region of chromosome 12. Such abnormalities also are found in dedifferentiated liposarcoma. Perhaps, genetic association is best characterized for myxoid liposarcoma. This represents a translocation between two chromosomes. In myxoid liposarcoma, the translocation is between chromosomes 12 and 16. The result is a gene called TLS-CHOP which is an oncogene. This particular translocation is highly specific for myxoid liposarcoma and therefore is diagnostic of this tumor [24].

Surgical curative resection with clear margins remains the mainstay of treatment except in cases of advanced liposarcoma with metastasis where palliative treatment can be given. But inspite of complete R0 resection, there is a high chance of recurrence particularly in pleomorphic variety. The role of radiotherapy is not well defined. Chemotherapy for high-grade liposarcoma has been tried with low success rates. Patient prognosis depends on histological subtype, grading, and tumor necrosis. The 5-year survival rate of patients who have undergone curative resection or radiation therapy is approximately 50% [25].

Table 2 is a list of previously reported cases [5–16]. 

Ours is the 13th case involving the left lobe for which left lateral lobectomy was done with HPE showing pleomorphic liposarcoma. The patient is still on followup and is tumor free till now. Based on the previous reports, The following predictions can be drawn about primary pleomorphic liposarcoma of liver.

- There is higher incidence in adult females with 7 cases as opposed to 3 in male adults and 3 involving children.
- There is no predilection for lobar involvement with 5 cases affecting each of the right and left lobes equally.
- It can present at any age ranging from 2-year baby to 86-year-old adult.
- Myxoid variety appears to be commonest as HPE studies have not been mentioned in all of the previous reported cases.
- Resection is the predominant mode of treatment as other modalities are still under evaluation.
- Myxoid liposarcoma carries poor prognosis.

## 4. Conclusion

Primary hepatic liposarcomas should be considered in differential diagnosis of liver tumors and computed tomography is helpful in evaluation of the tumor like any other visceral soft tissue sarcoma. Moreover, pleomorphic variety has a high metastatic potential compared to other histological variants. Neo adjuvant radiotherapy may be tried for high-grade tumors if diagnosed beforehand keeping in mind its occurrence. Although multimodality treatments combining chemoradiation have been put forward to achieve long-term survival, surgical resection still is the best means to achieve survival with other modalities serving a supplementary benefit.

## Figures and Tables

**Table 1:** 

Sl	Tumor	Abberation	Genes involved
(1)	Myxoid/round cell liposarcoma,	t(12;16)(q13;p11)	TLS-CHOP
(2)	Atypical lipomatous neoplasm/well-differentiated liposarcoma	Supernumerary ring chromosomes; giant marker chromosomes	Amplification of region 12q13-15, including MDM2, CDK4, HMGA2, SAS, GL1
(3)	Dedifferentiated liposarcoma	Supernumerary ring chromosomes; giant marker chromosomes	Amplification of region 12q14-15, including MDM2, CDK4, HMGA2, SAS, GL1
(4)	Pleomorphic liposarcoma	Complex alterations	UNKNOWN

(NCCN guidelines V.2.2009).

**Table 2:** 

Sl no	Age/sex	Location	Management	Followup	WHO subtype	Source
(1)	22/F	Right lobe	Right lobe hepatectomy	Survival 46 days	—	Wolloch et al., 1973 [5]
(2)	86/M	Capsule	Supportive treatment	—	Myxoid	Kim and Reyes, 1985 [6]
(3)	30/F	Left lobe	Left lobe hepatectomy	Tumor free for 10 month	Dedifferentiated	Kim et al., 1987 [7]
(4)	02/M	Hilum	Autopsy	—	—	Soares et al., 1989 [8]
(5)	03/M	Hilum	—	—	Myxoid	Wright et al., 1993 [9]
(6)	48/F	Hilum	Chemotherapy	—	—	Aribal and Berberoglu, 1993 [10]
(7)	54/F	Right and left lobes	Exploratory laparotomy with Biopsy	Postop. bleeding and death	Myxoid	Nelson et al., 2001 [11]
(8)	14/M	Right lobe	Right lobe hepatectomy	—	Round cell	Lu et al., 2003 [12]
(9)	61/F	Right lobe	Right lobe hepatectomy	Survival 27 months	Myxoid	Kuo et al., 2006 [13]
(10)	63/F	Left lobe	Left lobe hepatectomy	—	Well differentiated	Kim et al., 2007 [14]
(11)	21/F	Right lobe	Right lobe hepatectomy	Death 9 months after surgery	—	Nakhai and Motabar, 2007 [15]
(12)	64/M	Left lobe	Left lobe hepatectomy	Tumor free for 4 years	Myxoid	Lin et al., 2011 [16]
